# Longitudinally extensive myelitis as first presentation of renal cell carcinoma

**Published:** 2018-10-07

**Authors:** Behnaz Ansari, Ali Asghar Okhovat, Fereshteh Ashtari

**Affiliations:** 1 Department of Neurology, Alzahra Hospital, Isfahan University of Medical Sciences, Isfahan, Iran; 2 Department of Neurology, Sina Hospital, Tehran University of Medical Sciences, Tehran, Iran

**Keywords:** Intramedullary Spinal Cord Neoplasms, Transverse Myelitis, Renal Cell Carcinoma

Longitudinally extensive myelitis (LETM) is spinal cord inflammation involving three or more vertebral segments. The most common cause of LETM is neuromyelitis optica spectrum disorders (NMOSD) and other inflammatory demyelinative disease. Neuromelitis optica (NMO) is a demyelinating syndrome of the nervous system by attacks of optic neuritis and myelitis. Intramedullary tumors such as astrocytoma, ependymoma, and intravascular lymphoma are other causes of LETM, and intramedullary metastasis is very rare. However, development of progressive myelopathy in the absence of antecedent neurological symptoms and without signs indicating dissemination beyond the spinal cord is a diagnostic challenge, and rule out of other causes of myelitis should be done.^1^

There have been many reports of patients with demyelinating disorders mimicking spinal cord tumors,^2^^-^^5^ and on the contrary, we report a rare case of renal cell carcinoma (RCC) presenting by LETM without manifestation of primary tumor. 

A 70-year-old farmer without any other medical history was referred in October 2014 with right hand paresthesia and neck pain that radiated to shoulders. 

The first cervical magnetic resonance imaging (MRI) showed cervical spondylosis without abnormality in spinal cord parenchyma. The patient was treated for cervical spondylosis.

Two months later he noticed atrophy of first interosseous muscle and brain MRI was performed, which revealed a few small vessel ischemia and electromyography study showed motor neuron disease.

In May 2015, he was referred to our hospital with paraparesis (left more than right). At physical examination, mental examination was normal and paraparesis with a motor grade 3/5 in left and 4/5 in right side was detected without sphincteric problem.

The patient underwent whole spine MRI, which revealed an extensive intramedullary hyperintensity in T2-weighted MRI involving predominantly central spinal cord and extended until medulla location (Figure 1, A).

Routine investigations including hematology, biochemistry, electrocardiography (ECG), echocardiography, and chest X-ray showed no abnormality. The patient complained of dyspnea. LETM due to inflammatory demyelinated and primary spinal cord tumor were main differential diagnosis. High-dose methyl prednisolone was started and weakness and dyspnea was recovered dramatically after 5 days of treatment. Serum sample was referred for vasculitis tests and NMO antibody. Brain and cervical MRI with gadolinium and computed tomography (CT) scan of thorax and abdomen with contrast was done. After discontinue of corticosteroid, motor function in both lower extremities deteriorated without sphincter problem.

The second MRI of the cervical spine revealed an ill-defined T1 sequence isointense and T2 hyperintense intramedullary lesion extending from medulla to T2 levels with cord enlargement. There were two lesion enhancements (Figure 1, B-C). Abdominal CT scan showed large renal cell mass (Figure 1, D), and serum NMO-IgG was negative. However, the neurological features were highly suggestive of intramedullary lesion secondary to RCC. Radiotherapy was recommended by oncologist. After two weeks, before beginning treatment, the patient admitted for sign of sever hematuria and acute renal failure and unfortunately died of complications.

LETM is one of the common presentations of NMOSD. MRI of the spine is very helpful in diagnosing NMOSD. In acute phase, it typically shows LETM and gadolinium enhancement; in chronic stage, the enhancement and expansion resolve while the T2 signal persists, but the LETM regresses to less than three segments and breaks up in to shorter fragments.^1^

Many neoplastic lesions are misdiagnosis as intramedullary spinal cord myelitis such as NMOSD. More specifically, the fallowing misdiagnoses are made: infectious disorders, demyelination [multiple sclerosis (MS) and NMOSD], granulomatous disease, vascular lesions, and syringomyelia. 

**Figure 1:**
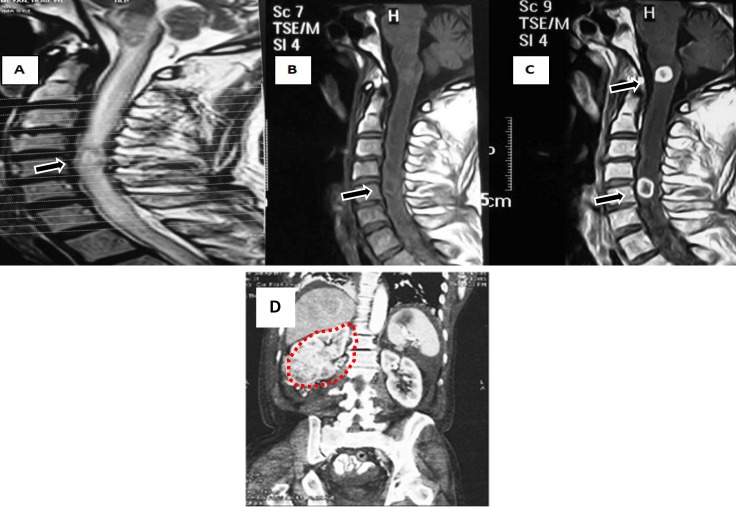
Magnetic resonance imaging (MRI) of the cervical spine; longitudinally extensive edema and fusiform cord enlargement extending from the medulla to T2 level. A) T2-sequence, sagittal cord MRI: Edema predominantly affecting the center of the spinal cord. B) FLAIR-sequence, sagittal brain MRI: nonspecific lesions and extending the cord lesion until medulla location. C) T1-sequence, sagittal brain MRI with contrast: two enhancement lesions with diffuse edema. D) Coronal computed tomographic image of the abdomen demonstrating a renal mass in right kidney (red dashed line)

It is difficult to distinguish these lesions from neoplastic spinal cord only by history and neurological examination. On brain MRI, most lesions are non-specific and asymptomatic, except for optic nerve enhancement in optic neuritis. The age onset of NMO ranges from childhood to late adulthood. The incidence tapering off after the fifth decade. However, there are many reports of very late onset of NMOSD.^3^

Otherwise, neoplastic diseases can cause LETM through intramedullary tumor or metastasis. Although intramedullary metastasis is rare, lung, breast, and RCCs are common underlying causes. So, it seems reasonable to consider intramedullary metastasis in patients with history and manifestation of neoplasm. On the other hand, tumors can cause LETM as paraneoplastic lesions before detection of malignancy, particularly RCC.^4^

RCC is unique among the genitourinary malignancies in that close to one third of affected patients show symptoms of a paraneoplastic syndrome in addition to central nervous system metastasis. Such as neuromyopathies have been described in cases of nonmetastatic RCC. Degrees of severity vary from nonspecific myalgias to bilateral phrenic nerve paralysis. There is evidence to suggest that some RCCs may secrete a substance that may mediate such neuromyopathies.^5^

We have to acknowledge limitation in this case due to lack of autopsy result. Actually, history of disease, age of patient, and antecedent neurological or systemic symptoms beyond the spinal cord were so helpful for diagnosis. This case report suggests that LETM may be a rare presenting manifestation of malignancy, which may be due to paraneoplastic etiology or intramedullary spinal cord metastasis. It is recommended that in an elderly patient with LETM, possibility of any underlying malignancy should be investigated, and the NMOSD be noticed as the second diagnosis.

## Notes:

**How to cite this article:** Ansari B, Okhovat AA, Ashtari F. Longitudinally extensive myelitis as first presentation of renal cell carcinoma. Iran J Neurol 2018; 17(4): 197-9.
